# Broadband Lamb Wave Trapping in Cellular Metamaterial Plates with Multiple Local Resonances

**DOI:** 10.1038/srep09376

**Published:** 2015-03-20

**Authors:** De-Gang Zhao, Yong Li, Xue-Feng Zhu

**Affiliations:** 1 Department of Physics, Huazhong University of Science and Technology, Wuhan, 430074, China; 2 Department of Physics, Hong Kong University of Science and Technology, Clear Water Bay, Hong Kong, China; 3 Innovation Institute, Huazhong University of Science and Technology, Wuhan, 430074, China

## Abstract

We have investigated the Lamb wave propagation in cellular metamaterial plates constructed by bending-dominated and stretch-dominated unit-cells with the stiffness differed by orders of magnitude at an ultralow density. The simulation results show that ultralight metamaterial plates with textured stubs deposited on the surface can support strong local resonances for both symmetric and anti-symmetric modes at low frequencies, where Lamb waves at the resonance frequencies are highly localized in the vibrating stubs. The resonance frequency is very sensitive to the geometry of textured stubs. By reasonable design of the geometry of resonant elements, we establish a simple loaded-bar model with the array of oscillators having a gradient relative density (or weight) that can support multiple local resonances, which permits the feasibility of a broadband Lamb wave trapping. Our study could be potentially significant in designing ingenious weight-efficient acoustic devices for practical applications, such as shock absorption, cushioning, and vibrations traffic, *etc*.

Over the past two decades, the propagation of acoustic waves in highly dispersive materials, such as phononic crystals and acoustic metamaterials, has been intensely studied due to the possibilities of existing acoustic band gaps^1^,^2^,^3^ as well as anomalous dispersions associated with Dirac-like cones^4^,^5^ and near zero^6^,^7^,^8^,^9^,^10^,^11^,^12^ or negative^13^,^14^ group velocities. Artificial materials with these intriguing properties have found myriad potential applications, such as acoustic cloaking^15^,^16^,^17^,^18^,^19^, vibration isolation^20^,^21^,^22^,^23^,^24^,^25^, wave guiding^26^,^27^, acoustic energy filtering and trapping^12^,^28^,^29^,^30^, *etc*. For the constituent material of phononic crystals and metamaterials with contrasted properties, it is normal that stiff material has a larger mass density than that of soft one. However, it is exceptional that one material and its allotropic form of comparable densities can have very different elastic properties due to the rather different configurations on the molecular level. In nature, there are two well-known allotropes of carbon: graphite and diamond^31^. In graphite, carbon atoms are bonded to form a two-dimensional (2D) network composed of hexagonal rings, and the resulting 2D sheets are loosely stacked via van der Waals forces. That weak bonding force between stacking sheets will lead to the crystalline instability. While in the more compact allotrope diamond, each carbon atom is bonded tetrahedrally to four other atoms, thus building up a topologically stable three-dimensional (3D) network that forms the most rigid material in nature to the best knowledge. Analogously, we can find a way to achieve ultralight metamaterials of widely varying elastic properties by evolving topologically stable and unstable cellular frameworks^32^,^33^,^34^,^35^. Exploration of elastic wave propagation in such composite cellular structures offers a new door to acoustic metamaterials with rich physics and useful functionalities, where ingenious weight-efficient acoustic devices can be designed for practical applications, such as shocking absorption, cushioning, and vibrations traffic, *etc*.

## Results

**Figure 1:**
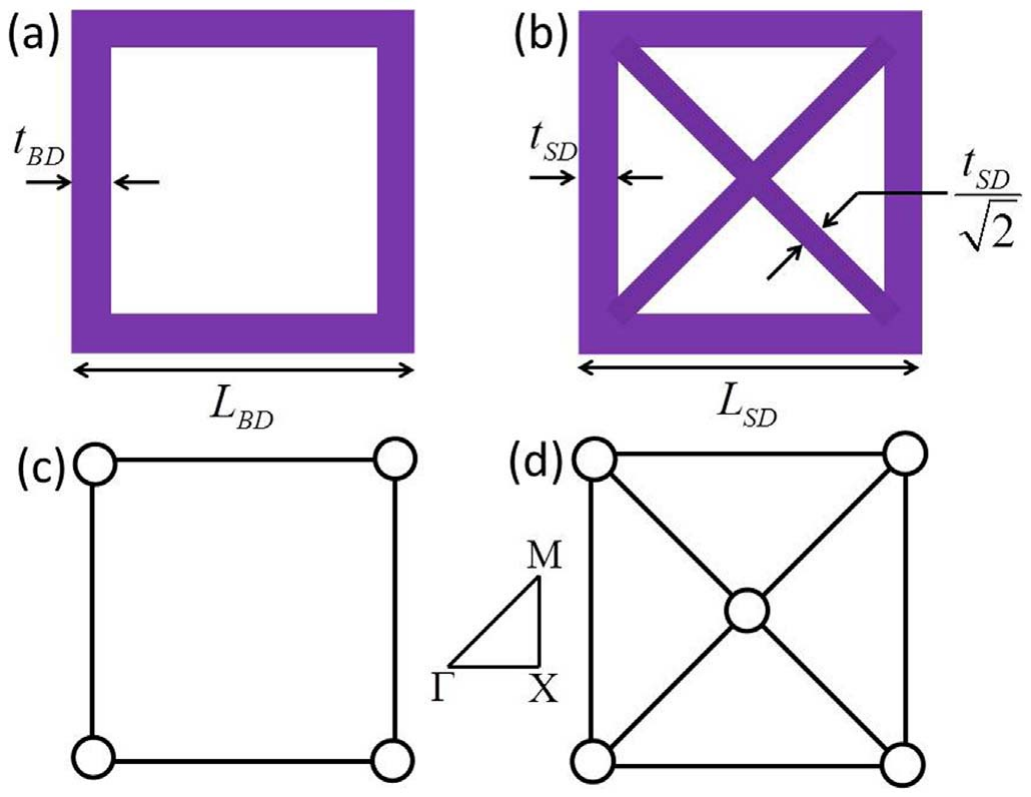
The schematic diagram of two typical unit-cells. Unit-cells of (a) the bending-dominated (BD) structure and (b) the stretch-dominated (SD) structure. Pin-jointed frameworks of (c) the BD structure and (d) the SD structure for analyzing the stability of cellular unit-cells. The inset is the irreducible Brillouin zone.

For simplicity, we only consider 2D cellular structures with two typical unit-cells which are named bending-dominated (BD) structure and stretch-dominated (SD) structure, respectively, as schematically shown in Figs. 1 (a) and 1(b). For the two square unit-cells, lengths of each side are *L_BD_*and *L_SD_*, and the thicknesses of bars are *t_BD_* and *t_SD_*, except for the additional crossing bars in the SD structured unit-cell with the thickness being 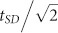. We choose steel as the constituent material for which the material density, longitudinal wave velocity, and transverse wave velocity are *ρ_s_* = 7850 kg/m^3^, *c_l_* = 6145 m/s, and *c_t_* = 3095 m/s, respectively. It needs to be mentioned that the fabrication of cellular steel frameworks can be made possibly by projection microstereolithography or 3D metal printing^35^. The relative densities of BD and SD structured unit-cells in Figs. 1(a) and 1(b) are concisely expressed as
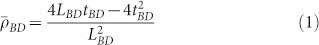
and
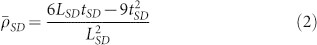
Here, the relative density is defined by the density of cellular structures over the density of steel, where the detailed derivations can be found in the *Supplementary Information*. In the following simulations, we set *L_BD_* = *L_SD_* = *L* = 2 mm as an example, with the thicknesses of bars *t_BD_* and *t_SD_* both tunable parameters for tailoring the relative densities of BD and SD structured unit-cells.

The BD and SD structured unit-cells can be further simplified into pin-jointed frameworks with locked joints, which are shown in Figs. 1(c) and 1(d), respectively. Their topological stability can be judged by a simple yet fundamental rule: Maxwell's stability criterion^35^:
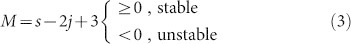
where *s* and *j* are the numbers of struts and joints. For the BD structured unit-cell [Fig. 1(c)], *M* = −1 < 0, according to Eq. (3). As a consequence, the unit-cell is topologically unstable, where bars in this structure will bend easily under the action of an external force. However, for the SD structured unit-cell [Fig. 1(d)], *M* = 1 > 0. The structure becomes both statically and kinematically determinate, indicating the SD structure is much stiffer than BD one in the prescribed directions due to the fact that the crossing bars can carry tension or compression when an external force is applied.

**Figure 2:**
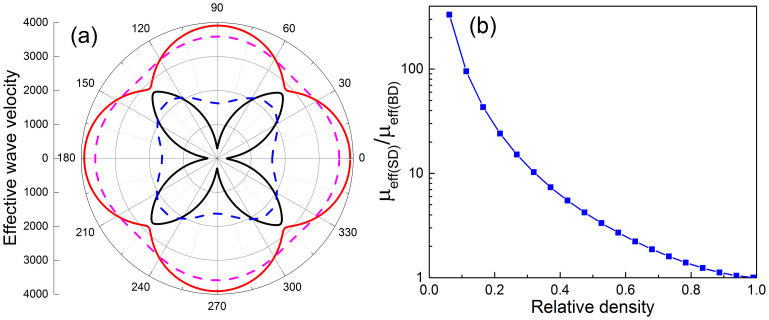
Effective mechanical properties of the cellular metamaterials. (a) The effective elastic wave velocities for cellular metamaterials of the BD structure (longitudinal: red solid line, transverse: black solid line) and the SD structure (longitudinal: pink dashed line, transverse: blue dashed line) along different directions, where the relative density is 0.19 for both cases. (b) The ratio of effective shear modulus of the SD structure to the BD structure versus the relative density of cellular metamaterials along the direction Γ-X.

In elastic mechanics, the stiffness of a solid material can be characterized by its elastic moduli or the associated elastic wave velocities. In order to investigate the effective mechanical properties of BD and SD structures in the long wavelength condition, we have calculated the band structures of 2D periodic superlattices with BD and SD structured unit-cells, and further obtained the effective longitudinal and transverse wave velocities in all directions by using ***ν****_l_*_(*t*)_ = ***ω****_l_*_(*t*)_/***k****_l_*_(*t*)_, where ***k****_l_*_(*t*)_ → 0. During the calculation, the relative densities of BD and SD structures are set to be 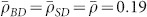, while the magnitude of Bloch wave vector |***k****_l_*_(*t*)_| is 0.02*π*/*L*. It is noted that the Bloch wavelength *λ_l_*_(*t*)_ = 2*π*/|***k****_l_*_(*t*)_| = 100*L* is much larger than the size of unit-cells (side length: *L*). Therefore, the BD and SD structures can be regarded as homogeneous metamaterials for the propagating elastic waves at low frequencies. The effective longitudinal and transverse wave velocities of BD and SD structures in all directions are displayed in Fig. 2(a), where the 0°and 45° represent Γ-X and Γ-M directions, respectively. It clearly reveals that the wave velocities in BD structured metamaterial have much greater variation along with different directions than those in SD one. It denotes the BD structure is much more anisotropic than the SD structure. Specifically, in the Γ-X direction, the transverse wave velocity of SD structured metamaterial is over one order of magnitude larger than that of BD structured one, indicating the BD structured metamaterial is much more flexible in this case. We then set our focus on the Γ-X direction and map out the relation between the ratio of effective shear modulus *μ_eff_*_(*SD*)_/*μ_eff_*_(B*D*)_. (here 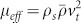 for both BD and SD structured metamaterials) and relative density in Fig. 2(b). It is interesting to find out that the contrast of *μ_eff_* will rapidly increase as the relative density decreases. For example, at an ultralow relative density 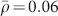, the ratio of *μ_eff_* between SD and BD structured metamaterials can be as large as ~ 332. These two cellular metamaterials with different topologies exhibit a remarkable stiffness contrast at low densities, which provides a unique route towards the next generation of ultralight acoustic devices for various wave manipulation applications.

**Figure 3:**
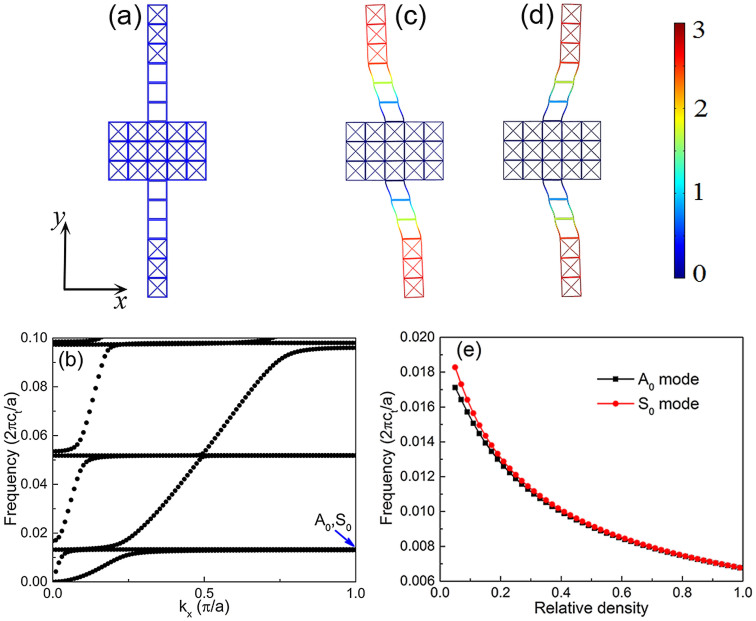
Eigenmodes and eigenfields of cellular metamaterial plates with periodic composite stubs. (a) One unit-cell of cellular metamaterial plates with a pair of stubs, where the lattice constant *a* = 5*L* = 10 mm. The unit-cells are periodically arrayed in *x*-direction, whose band structure is calculated in (b). For the lowest locally resonant eigenmodes marked by an arrow in (b), the displacement eigenfield distributions with scaled deformations are shown in (c): anti-symmetry mode (A_0_) and (d): symmetry mode (S_0_), respectively. (e) The resonance frequencies of A_0_ mode and S_0_ mode versus the relative density of SD caps in the composite stubs. Here and hereafter, the frequency is normalized by 2*πc_t_*/*a*.

Taking advantage of the great contrast of effective shear moduli in the Γ-X direction of metamaterials with SD and BD structured unit-cells, we can construct locally resonant elements by purposely assembling these two typical unit-cells. The resonant element is shown schematically in Fig. 3(a), which is featured by 5 × 3 arrayed SD structured unit-cells in the substrate, with a pair of composite stubs made of a 1 × 3 SD structured cap on a 1 × 3 BD structured base deposited on both sides of the substrate. The geometrical parameters of SD and BD structured unit-cells are the same as those presented in Fig. 2(a). Since the SD structured cap is much less flexible than the BD structured base, this composite stub can be regarded as a resonator where a stiff load is fixed at the end of a soft bar, *viz*. a loaded-bar system^10^. Due to the strong coupling of Lamb waves propagating in the substrate and the local resonances of the composite stub pairs, several flat bands in low frequencies can be clearly observed in the band structure as shown in Fig. 3(b). All these flat bands are the mixtures of two degenerate resonant modes: anti-symmetry modes (A) and symmetry modes (S).

To give an intuitive understanding of the local resonances in the system, we have plotted in Figs. 3(c) and 3(d) the displacement field distributions of eigenmodes [marked by A_0_ and S_0_ in Fig. 3(b)] at the lowest flat band near the edge of the Brillouin zone to present how the composite stubs vibrate under those conditions. The eigenfield distribution of A_0_ mode shows that the composite stubs on two sides of the substrate plate are vibrating out-of-phase. While for S_0_ mode, the vibrations of two stubs are in-phase. For each vibrating composite stub, it gives a remarkable resemblance to the first-order bending mode in a loaded-bar system. The phase differences between the two degenerate resonant modes are actually originated from the vibration features of anti-symmetry and symmetry Lamb wave modes^10^. Since the SD structured cap is viewed as the mass load, its density or weight will greatly influence the resonance frequency. The dependence of resonance frequency on the relative density of SD structured caps is sketched in Fig. 3(e). It distinctly reveals that the resonance frequency is very sensitive to the density of SD caps, especially in the lower density situation. In the lower density region (<0.2), the resonance frequencies of A_0_ mode and S_0_ mode have a small discrepancy. However, as the relative density of the caps increases (or becomes heavier), these two modes will eventually be degenerate. Thanks to the zero group velocities of the flat bands as well as its sensibility to the resonator geometries, a judicious design of a tailored resonant element array is possible to achieve Lamb wave trapping, with some intriguing properties: trapping vibrations in a deep subwavelength scale, in a broad frequency band, and for both anti-symmetric and symmetric modes.

**Figure 4:**
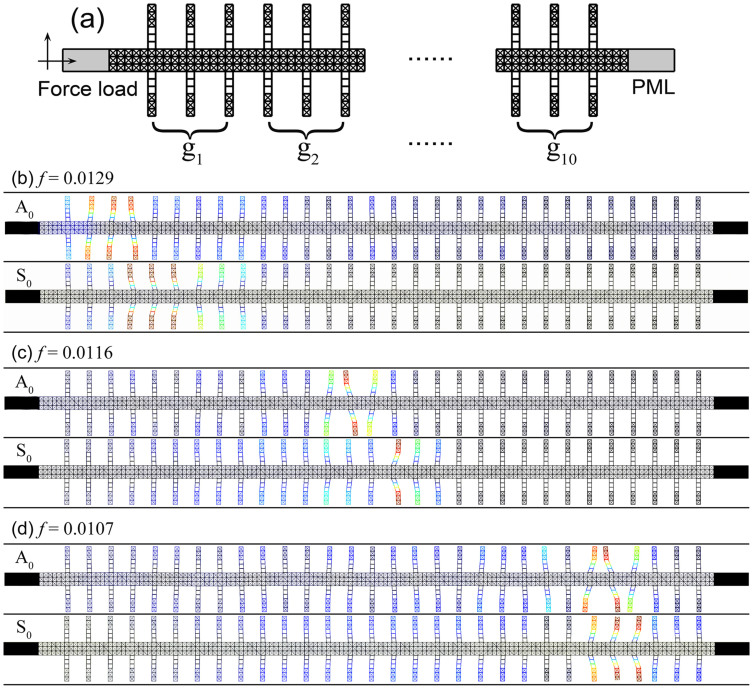
The numerical demonstration of Lamb wave rainbow trapping. (a) A schematic view of the cellular metamaterial plate for broadband Lamb wave trapping. (b)–(d) Displacement field distributions for A_0_ mode and S_0_ mode stimulations of three selected driving frequencies, respectively.

In previous works, many plate systems with stubbed surfaces were employed to achieve broadband Lamb wave^23^ or surface wave trapping^12^. In our case, the cellular metamaterial plate for broadband Lamb wave trapping is shown in Fig. 4(a), which consists of an array of resonant elements in Fig. 3(a) with tailored properties along the *x*-direction. The resonant elements, grouping in threes, have a linearly increasing relative density for the SD structured caps. The number of groups is 10 (the total number of resonant elements is 30) and the relative density of SD structured caps in the group *i* is 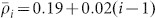. According to the curves in Fig. 3(e), our system can trap Lamb waves in the frequency region from ~0.0103 to ~0.0133. We note that this trapping frequency bandwidth can be further broadened by adding more tailored resonant elements. The displacement field distributions of three selective frequencies, *viz*. 0.0129, 0.0116, and 0.0107, are numerically calculated and sketched, for both A_0_ mode and S_0_ mode in Figs. 4(b) to 4(d). According to Fig. 3(e), when the resonance frequencies of A_0_ mode and S_0_ mode are set to be 0.0129, the relative densities of corresponding SD structured caps should be about 0.2 and 0.21, respectively. Therefore, we can observe that A_0_ mode and S_0_ mode are trapped in the left part of our system with some differences in the trapping position. In a similar way, for the frequency of 0.0116 (the relative density of corresponding SD structured caps is approximately 0.27), both A_0_ mode and S_0_ mode will be trapped in the middle part of the system with a smaller difference in the trapping position. When the frequency decreases further down to 0.0107 (the relative density of corresponding SD structured caps is approximately 0.33), Lamb waves are trapped in the right part of the system, while A_0_ mode and S_0_ mode are localized nearly at the same positions. The results in Figs. 4(b)–4(d) are in good agreement with the theoretical predictions in Fig. 3(e). From the above, we demonstrate that a broadband Lamb wave trapping in a cellular metamaterial plate is physically realizable by purposely introducing multiple local resonances through tailoring the weight of stub caps, let alone its unprecedented weight efficiency.

## Discussion

In summary, we have investigated cellular metamaterials of two typical structures: BD structure and SD structure, which possess superior bulk-scale elastic properties, such as greatly contrasted stiffness at an ultralow density. By purposely arranging BD and SD structured unit-cells, we have developed a prototype of cellular metamaterial plates with composite textured stubs deposited on the surfaces for producing strong local resonances. Since the resonance frequency is very sensitive to the relative density (or weight) of the stub caps, a broadband Lamb wave trapping is therefore realizable by tailoring the geometry of resonant elements. In this paper, we only focus on the lowest flat bands for the Lamb wave trapping. At the lowest flat bands, the motion of resonant stubs is mainly a shear vibration, for which the shear modulus *μ_eff_* plays a dominant role in local resonances. Therefore we can ignore the effects of the other Lamé coefficient *λ_eff_*. As a matter of fact, we can also achieve Lamb wave trapping at the flat bands of higher frequencies. In this case, more complicated compression and extension will kick in for the resonant stubs, so that *λ_eff_* also needs to be considered. Further studies should be performed for exploring trapping properties of metamaterials with 3D cellular frameworks (see the *Supplementary Information*), as well as the sample fabrication and experimental characterization. Our system could find its significance in designing myriad ultralight devices for elastic wave guiding and filtering, as well as acoustic energy harvesting.

## Methods

Throughout this paper, numerical simulations are carried out by the finite element solver in commercial software COMSOL Multiphysics with the help of a high performance computing cluster. In Fig. 2, we use BD and SD structures as the unit cells and calculate their band structures. Then we employ the linear dispersion lines in the long wave approximation to calculate their effective wave velocities and shear moduli. In Fig. 4, a uniform steel layer is added at the left side of trapping structure and an external normal/tangential oscillating stimulus is loaded on the left boundary to provide an input of longitudinal/transverse waves. The amplitude of force load per unit area is set to be 1 Pa. A perfectly matched layer (PML) is added at the right side to prevent reflections.

## Author Contributions

D.G.Z. and X.F.Z. carried out the numerical simulations and theoretical analysis. X.F.Z., D.G.Z. and Y.L. contributed in the discussion of theoretical analysis and wrote the paper. X.F.Z. conceived and supervised the study.

## Supplementary Material

Supplementary Information Supplementary Information

## References

[b1] Martinez-Sala R. *et al.* Sound attenuation by sculpture. Nature 378, 241 (1995).

[b2] Sigalas M. M. & Ecomonou E. N. Elastic and acoustic wave band structure. J. Sound Vib. 158, 377 (1992).

[b3] Kushwaha M. S., Halevi P., Dobrzynski L. & Djafari-Rouhani B. Acoustic band structure of periodic elastic composites. Phys. Rev. Lett. 71, 2022 (1993). 10054563 10.1103/PhysRevLett.71.2022

[b4] LiuF. M., HuangX. Q. & ChanC. T. Dirac cones at 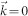 in acoustic crystals and zero refractive index acoustic materials. Appl. Phys. Lett. 100, 071911 (2012).

[b5] Lu J. Y. *et al.* Dirac cones in two-dimensional artificial crystals for classical waves. Phys. Rev. B 89, 134302 (2014).

[b6] Liu Z. *et al.* Locally resonant sonic materials. Science 289, 1734 (2000). 10976063 10.1126/science.289.5485.1734

[b7] Zhu X. F. Effective zero index in locally resonant acoustic material. Phys. Lett. A 377, 1784 (2013).

[b8] Pennec Y., Djafari-Rouhani B., Larabi H., Vasseur J. O. & Hladky-Hennion A. C. Low-frequency gaps in a phononic crystal constituted of cylindrical dots deposited on a thin homogeneous plate. Phys. Rev. B 78, 104105 (2008).

[b9] Wu T. C., Wu T. T. & Hsu J. C. Waveguiding and frequency selection of Lamb waves in a plate with a periodic stubbed surface. Phys. Rev. B 79, 104306 (2009).

[b10] Oudich M., Li Y., Assouar B. M. & Hou Z. L. A sonic band gap based on the locally resonant phononic plates with stubs. New J. Phys. 12, 083049 (2010).

[b11] Rupin M., Lemoult F., Lerosey G. & Roux P. Experimental Demonstration of Ordered and Disordered Multiresonant Metamaterials for Lamb Waves. Phys. Rev. Lett. 112, 234301 (2014). 24972210 10.1103/PhysRevLett.112.234301

[b12] Zhu J. *et al.* Acoustic rainbow trapping. Sci. Rep. 3, 1728 (2013).

[b13] Yang S. X. *et al.* Focusing of sound in a 3D phononic crystal. Phys. Rev. Lett. 93, 024301 (2004). 15323920 10.1103/PhysRevLett.93.024301

[b14] Lu M. H. *et al.* Negative birefraction of acoustic waves in a sonic crystal. Nature Mater. 6, 744 (2007). 17721539 10.1038/nmat1987

[b15] Zhu X. F., Liang B., Kan W. W., Zou X. Y. & Cheng J. C. Acoustic cloaking by a superlens with single-negative materials. Phys. Rev. Lett. 106, 014301 (2011). 21231745 10.1103/PhysRevLett.106.014301

[b16] Zhang S., Xia C. & Fang N. Broadband acoustic cloak for ultrasound waves. Phys. Rev. Lett. 106, 024301 (2011). 21405230 10.1103/PhysRevLett.106.024301

[b17] Popa B. I., Zigoneanu L. & Cummer S. A. Experimental acoustic ground cloak in air. Phys. Rev. Lett. 106, 253901 (2011). 21770640 10.1103/PhysRevLett.106.253901

[b18] Stenger N., Wilhelm M. & Wegener M. Experiments on elastic cloaking in thin plates. Phys. Rev. Lett. 108, 014301 (2012). 22304261 10.1103/PhysRevLett.108.014301

[b19] Zhu X. F., Ramezani H., Shi C., Zhu J. & Zhang X. PT-symmetric acoustics. Phys. Rev. X 4, 031042 (2014).

[b20] Tanaka Y. & Tamura S. I. Surface acoustic waves in two-dimensional periodic elastic structures. Phys. Rev. B 58, 7958 (1998).

[b21] Vasseur J. O., Deymier P. A., Djafari-Rouhani B., Pennec Y. & Hladky-Hennion A. C. Absolute forbidden bands and waveguiding in two-dimensional phononic crystal plates. Phys. Rev. B 77, 085415 (2008).

[b22] Zhu X. F. Acoustic waves switch based on meta-fluid phononic crystals. J. Appl. Phys. 112, 044509 (2012).

[b23] Assouar M. B., Senesi M., Oudich M., Ruzzene M. & Hou Z. L. Broadband plate-type acoustic metamaterial for low-frequency sound attenuation. Appl. Phys. Lett. 101, 173505 (2012).

[b24] Oudich M. *et al.* Experimental evidence of locally resonant sonic band gap in two-dimensional phononic stubbed plates. Phys. Rev. B 84, 165136 (2011).

[b25] Lai Y., Wu Y., Sheng P. & Zhang Z. Q. Hybrid elastic solids. Nature Mater. 10, 620 (2011). 21706010 10.1038/nmat3043

[b26] Lemoult F., Kaina N., Fink M. & Lerosey G. Wave propagation control at the deep subwavelength scale in metamaterials. Nature Phys. 9, 55 (2013).

[b27] Otsuka P. H. *et al.* Broadband evolution of phononic-crystal-waveguide eigenstates in real- and k-spaces. Sci. Rep. 3, 3351 (2013). 24284621 10.1038/srep03351 PMC3842087

[b28] Huang Z. G. Silicon-based filters, resonators and acoustic channels with phononic crystal structures. J. Phys. D: Appl. Phys. 44, 245406 (2011).

[b29] Ding H. X., Shen Z. H., Ni X. W. & Zhu X. F. Multi-splitting and self-similarity of band gap structures in quasi-periodic plates of Cantor series. Appl. Phys. Lett. 100, 083501 (2012).

[b30] Zhao D. G., Ye Y. T., Xu S. J., Zhu X. F. & Yi L. Broadband and wide-angle negative reflection at a phononic crystal boundary. Appl. Phys. Lett. 104, 043503 (2014).

[b31] Mounet N. & Marzari N. First-principles determination of the structural, vibrational and thermodynamic properties of diamond, graphite, and derivatives. Phys. Rev. B 71, 205214 (2005).

[b32] Zheng X. *et al.* Ultralight, ultrastiff mechanical metamaterials. Science 344, 1373 (2014). 24948733 10.1126/science.1252291

[b33] Meza L. R., Das S. & Greer J. R. Strong, lightweight, and recoverable three-dimensional ceramic nanolattices. Science 345, 1322 (2014). 25214624 10.1126/science.1255908

[b34] Guest S. D. Tensegrities and rotating rings of tetrahedra: a symmetry viewpoint of structural mechanics. Philos. Trans. R. Soc. Lond. A 358, 229 (2000).

[b35] Deshpande V. S., Ashby M. F. & Fleck N. A. Foam topology: bending versus stretching dominated architectures. Acta Mater. 49, 1035 (2001).

