# Supplementary material for: Broadband Lamb Wave Trapping in Cellular Metamaterial Plates with Multiple Local Resonances
Source: Sci Rep. 2015 Mar 20;5:9376. doi: 10.1038/srep09376 (PMC4366801; doi:10.1038/srep09376)
Supplement: Supplementary Information [file srep09376-s1.pdf]

# Broadband Lamb Wave Trapping in Cellular Metamaterial Plates with Multiple Local Resonances

De-Gang Zhao[1,2], Yong Li[2], and Xue-Feng Zhu[1,3,a)]

[1]Department of Physics, Huazhong University of Science and Technology, Wuhan, 430074, China

[2]Department of Physics, Hong Kong University of Science and Technology, Clear Water Bay, Hong Kong, China

[3]Innovation Institute, Huazhong University of Science and Technology, Wuhan, 430074, China

* Corresponding Author: *xfzhu@hust.edu.cn*

# Supplementary Information

## I. The design of three-dimensional (3D) cellular structures.

In section I, we will briefly present the design of 3D bending-dominated (BD) structure and stretch-dominated (SD) structure. 3D BD and SD structured unit-cells are schematically shown in Figs. S1(a) and S1(b). The BD structured unit-cell consists of solid struts surrounding a void cubic space. $L_{BD}$ is the cell size and $t_{BD}$ is the thickness of the cell edges. The SD structured unit-cell is constructed by adding extra crossing bars as shown in Fig. S1(b). $L_{SD}$ is the length of the cell edges and $t_{SD}$ is the thickness of each bar. The relative densities of the 3D BD and SD structured unit-cells can be calculated by

$$\bar{\rho}_{BD} = \frac{12 L_{BD} t_{BD}^2 - 16 t_{BD}^3}{L_{BD}^3}, \tag{S1}$$

and

$$\bar{\rho}_{SD} = \frac{\left(15 + 12\sqrt{2}\right) L_{SD} t_{SD}^2 - \left(36 + 24\sqrt{2}\right) t_{SD}^3}{L_{SD}^3}. \tag{S2}$$

which will be proven in Section II. The 3D BD and SD structured unit-cells can be simplified into pin-jointed frameworks with locked joints, which are shown in Figs. S1(c) and S1(d), respectively. Their topological stability can be judged by Maxwell's stability criterion in 3D case[1]:

$$M = s - 3j + 6 \begin{cases} \geq 0\text{ , stable} \\ < 0\text{ , unstable} \end{cases} \tag{S3}$$

where $s$ and $j$ are the numbers of struts and joints, respectively. For the 3D BD structured unit-cell in Fig. S1(c), $s = 12$ , $j = 8$ , then $M = -6 < 0$ . As a consequence, the unit-cell is topologically unstable, where the bars in this structure will bend easily under the action of an external force. However, for the 3D SD structured unit-cell in Fig. S1(d), $s = 42$ , $j = 15$, then $M = 3 > 0$. The structure becomes both statically and kinematically determinate, indicating the SD structure is much stiffer than BD one in the prescribed directions due to the fact that the crossing bars can carry tension or compression when an external force is applied.

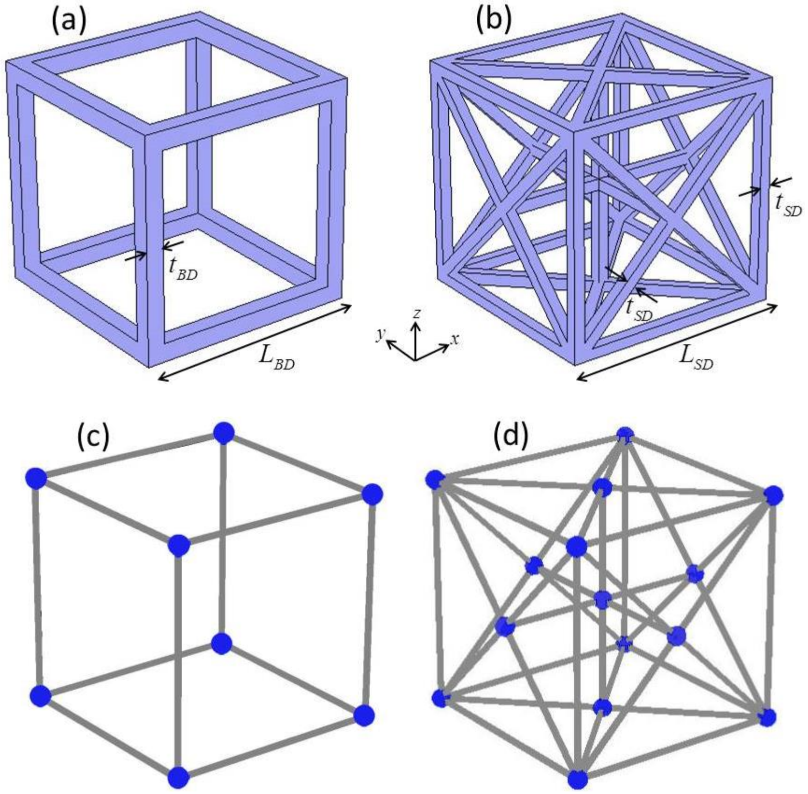


**Figure S1| Schematic diagrams of two typical 3D unit-cells.** Unit-cells of (a) the BD structure and (b) the SD structure. Pin-jointed frameworks of (c) the BD structure and (d) the SD structure for analyzing the stability of the 3D unit-cells.

In Fig. S2, we investigate the band structures and effective elastic properties of 3D BD and SD structured metamaterials in the long wavelength condition. In the calculation, we set $L_{BD} = L_{SD} = L = 2\text{mm}$ and $\bar{\rho}_{BD} = \bar{\rho}_{SD} = \bar{\rho} = 0.104$ ( $t_{BD} = 0.1L$ , $t_{SD} = 0.0613L$ ) as an example. For simplicity, we only consider the propagation of elastic waves in the *xy*-plane. Figs. S2(a) and S2(b) display the lowest bands in the $\Gamma - \mathrm{X}$ and $\Gamma - \mathrm{M}$ directions of the irreducible Brillouin zone. In the long wavelength condition, the bands can be categorized into in-plane longitudinal P mode band, in-plane transverse SV mode band, and out-of-plane transverse SH mode band. In the deep subwavelength regime as marked by the dashed boxes in Figs. S2(a) and S2(b), the dispersion curves turn into straight lines and their slopes can be regarded as the effective wave velocities. In Fig. S2(d), we present the relation between the ratio of effective shear modulus $\mu_{eff\,(SD)} / \mu_{eff\,(BD)}$ and relative density in the $\Gamma - \mathrm{X}$ direction. We observe that the curve in Fig. S2(d) is much steeper than the one in Fig. 2(b), indicating that the shear modulus contrast $\mu_{eff\,(SD)} / \mu_{eff\,(BD)}$ in 3D case is more sensitive to the relative density than that of 2D case.

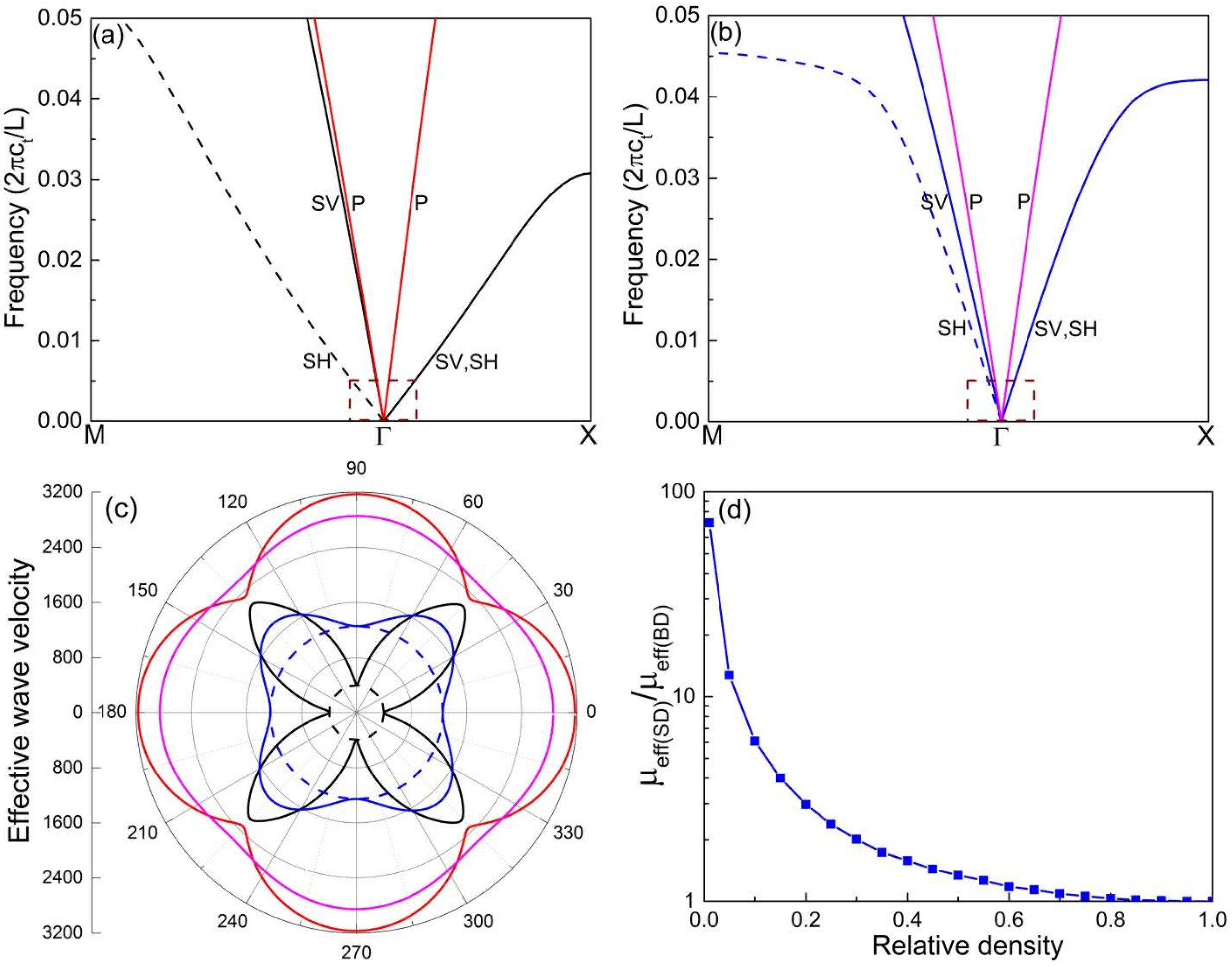


**Figure S2| Effective mechanical properties of 3D cellular metamaterials.** (a) and (b) are the band structures of cubic lattices of BD and SD structured unit-cells in low frequency region. We consider the Bloch waves propagating in the *xy*-plane for simplicity. In (a), solid red line, solid black line, and dashed black line represent the P mode band, SV mode band, and SH mode band, respectively. In (b), solid pink line, solid blue line, and dashed blue line represent the P mode band, SV mode band, and SH mode band, respectively. (c) depicts the effective elastic wave velocities for the 3D BD and SD structured metamaterials along different directions in the *xy*-plane, where the relative density is 0.104 for both cases. There is a one-to-one correspondence

between the lines in (a), (b) and those in (c) in terms of the color and style. (d) The ratio of effective shear modulus of SD structure to BD structure versus the relative density of 3D cellular metamaterials along the direction $\Gamma - \mathrm{X}$.

## II. The derivation of relative densities.

The relative density is the ratio of the density of cellular metamaterial to the density of the solid of which it is made, *i.e.* steel. Geometrically, it is equal to the volume fraction of solid in the metamaterial.

In 2D case, the relative density of BD structured unit-cells in Fig. 1(a) is

$$\bar{\rho}_{BD} = \frac{L_{BD}^2 - \left(L_{BD} - 2t_{BD}\right)^2}{L_{BD}^2} = \frac{4L_{BD}t_{BD} - 4t_{BD}^2}{L_{BD}^2}, \tag{S4}$$

and that of SD structured unit-cells in Fig. 1(b) is

$$\bar{\rho}_{SD} = \frac{L_{SD}^2 - \left(L_{SD} - 2t_{SD} - 2\cdot\dfrac{t_{SD}}{2}\right)^2}{L_{SD}^2} = \frac{6L_{SD}t_{SD} - 9t_{SD}^2}{L_{SD}^2}, \tag{S5}$$

In 3D case, the relative density of BD structured unit-cells in Fig. S1(a) is

$$\bar{\rho}_{BD} = \frac{L_{BD}^3 - \left(L_{BD} - 2t_{BD}\right)^3 - 6t_{BD}\left(L_{BD} - 2t_{BD}\right)^2}{L_{BD}^3} = \frac{12L_{BD}t_{BD}^2 - 16t_{BD}^3}{L_{BD}^3}, \tag{S6}$$

and that of SD structured unit-cells in Fig. S1(b) is

$$\bar{\rho}_{SD} = \frac{L_{SD}^3 - \left(L_{SD} - 2t_{SD}\right)^3 + 3t_{SD}^2\left(L_{SD} - 2t_{SD}\right) + 2t_{SD}^3 - 6t_{SD}\left(L_{SD} - 2t_{SD} - \sqrt{2}t_{SD}\right)^2}{L_{SD}^3} = \frac{\left(15 + 12\sqrt{2}\right)L_{SD}t_{SD}^2 - \left(36 + 24\sqrt{2}\right)t_{SD}^3}{L_{SD}^3} \quad \text{(S7)}$$


1. Calladine, C. R. Theory of Shell Structures, *Cambridge University Press*, Cambridge, UK, 1983.
